# Reply to Abid et al. Comment on “Aşır et al. Investigation of Vitamin D Levels in Men with Suspected Infertility. *Life* 2024, *14*, 273”

**DOI:** 10.3390/life14070914

**Published:** 2024-07-22

**Authors:** Fırat Aşır, Tuğcan Korak, Zuhal Çankırı

**Affiliations:** 1 Department of Histology and Embryology, Medical Faculty, Dicle University, 21280 Diyarbakır, Turkey; 2 Department of Medical Biology, Medical Faculty, Kocaeli University, 41001 Kocaeli, Turkey; tugcankorak@gmail.com

**Keywords:** UV radiation, dietary, Vitamin D, male infertility

## Abstract

In response to the insightful comments made by Dr. Abid et al. on our article “Investigation of Vitamin D Levels in Men with Suspected Infertility”, we address several key points concerning the generalizability and methodology of our study. Dr. Abid et al.’s critique primarily focused on the single-center nature of our research, regional variations in ultraviolet (UV) exposure, dietary factors affecting vitamin D levels, and the sample size of our study. We discuss the inherent value and controlled environment of single-center studies while acknowledging the need for multi-center validation. Additionally, we explain our consideration of sun exposure and dietary intake in our analysis, and recognize the importance of larger, more diverse studies to strengthen our findings. Our response aims to clarify these aspects and emphasize the significance of vitamin D in male infertility, encouraging further research in this field.

## 1. Introduction

We appreciate the opportunity to address the comments made by Dr. Abid et al. regarding our article, “Investigation of Vitamin D Levels in Men with Suspected Infertility” [1]. Our article explores the relationship between vitamin D levels and male infertility, focusing on sperm motility and functionality. Conducted on 306 male participants, our research involved the collection and analysis of semen samples, categorizing participants into groups based on their vitamin D levels (low, <20 ng/mL and high, ≥20 ng/mL). The study also employed bioinformatical databases to identify potential molecular targets and mechanisms in male infertility. Vitamin D has important roles in human physiology and vitamin D receptors (VDRs) are found in 60 types of cells [2]. The expression of VDR in the testicular tissues suggests that vitamin D has a role in testicular function and male fertility [3]. In a review on sperm function and vitamin D by Cito et al. [4], the authors posited that vitamin D has beneficial effects on semen parameters and sperm motility. However, they also noted that experimental studies have produced controversial results regarding the relationship between vitamin D and sperm parameters. The authors emphasized the need for future studies to elucidate the precise role of vitamin D in sperm function and parameters in both fertile and infertile men. Dr. Abid et al. claimed that our results contradict those of Banks et al. [5]. Upon examining the findings of Banks et al.’s study, we noted that their focus was primarily on the relationship between ethnicity and a few semen parameters (sperm concentration, morphology, and DNA fragmentation). They grouped subjects of different ethnicities together for analysis with vitamin D levels. In contrast, our study exclusively analyzed sperm morphology and semen parameters in detail for the Turkish population. Therefore, differences in specific parameters may exist due to the distinct focus and population of our study.

## 2. Use of Single-Center Study Database

Dr. Abid et al. raised concerns about the generalizability of our findings due to the single-center nature of our study. Multi-center studies can offer a broader perspective, larger sample size, data sharing across different centers, and enhanced generalizability and conversion of clinical data for daily medical practice [6]. Although multi-center studies are important for clinical and public health research [7], the data from these studies may differ due to different factors. For example, the drug dosage, timing, and application can be varied. For invasive interventions, data may be unreliable, since each physician can use different criteria for the same patients [8]. Another issue is that the implementation of data and statistical challenges can compromise the validity of a study [9]. The results of multi-center studies can be misleading, for example, because gene polymorphisms can be variable and can affect the predictability of findings [10]. Single-center studies also hold significant value, particularly when they are conducted with rigorous controls and detailed methodological frameworks. They are relatively cheaper and easier to conduct and design [11]. Single-center studies allow flexibility for clinicians and scientists to develop innovative treatments, serving as a crucial source of new therapeutic concepts. Our study was designed to control for a variety of confounding factors within a single, consistent environment, providing a high level of internal validity. We analyzed numerous sperm parameters and hormone levels in detail. The center from which we retrieved data is a large-scale hospital that serves at least 15 different cities across Turkey. This broad catchment area enhances the randomness of our sample and reduces the risk of data bias.

## 3. Regional Variations in Sun Exposure

Recent studies showed an association between UV radiation and male infertility. It is known that male fertility is affected by prolonged exposure to heat, hazardous chemicals, pesticides, and radiation, especially ionizing radiation, since radiation causes DNA damage and genomic instability [12]. In an in vitro study by Rajput et al. [13], sperm from infertile males were susceptible to UV exposure compared to those from fertile males in terms of sperm DNA damage, sperm viability, and nuclear chromatin decondensation. Another study also demonstrated similar results, claiming that infertile males showed a higher percentage of immature sperm compared to fertile males after UV exposure [14]. Vitamin D is acquired through diet and synthesized in the skin via ultraviolet B (UV-B) radiation from sun exposure [15]. Vitamin D deficiency is a common problem, with a global prevalence of 15.7% [16]. In the region from which our study population comes, the prevalence of vitamin D deficiency is about 36% [17]. Considering these findings, it is evident that while sunlight exposure is essential for the synthesis of vitamin D in the skin, a balance must be maintained. Excessive UV exposure can adversely affect spermatogenesis, as prolonged exposure to UV radiation has been shown to cause DNA damage and genomic instability in sperm. Moreover, the impact of UV radiation on male fertility can vary based on regional and environmental differences. The critique regarding variations in UV exposure and its impact on vitamin D levels is well formed. Our study population was located in a region with relatively consistent sunlight exposure, which we controlled for by collecting data year-round and accounting for seasonal variations. Future research could benefit from including diverse geographic locations to examine how regional differences in sun exposure influence vitamin D levels and infertility outcomes.

## 4. Dietary Factors

Dr. Abid et al. suggested that our study should have considered dietary factors more comprehensively. We are aware of the importance of dietary factors in fertility. A recent study by Erdoğan-Gövez et al. [18] indicated that the primary nutrition of Turkish people is meat-based, while vegetable-based foods are among the least consumed by males aged 15 to 64 years. According to a report by the Turkish Health Ministry [19], 7.5% of people consume some type of meat (cow, sheep, fish, or turkey) daily, and 36% consume eggs daily. Another study showed that less than 5% of people in Turkey adhere to vegan or vegetarian diets [20]. Scientific data indicate that both males and females in Turkey predominantly consume meat or its derivatives and animal-based meals. Although this led us to exclude dietary habits from our analysis, further studies should incorporate more robust dietary evaluations to isolate the effects of vitamin D in the diet.

## 5. Role of Endocrine-Disrupting Chemicals (EDCs)

Endocrine-disrupting chemicals (EDCs) such as bisphenol A, phthalates, pesticides, and other environmental chemicals are shown to cause adverse impacts on male fertility [21]. Sperm parameters including production, quality and morphology, motility, concentration, volume, and morphology were reduced after EDC exposure in rodents [22,23,24,25]. Similarly, Faure et al. [26] suggested that prolonged exposure to EDCs caused negative impacts on sperm quality, motility, and morphology. It should also be remembered that humans can be exposed to many chemicals and environmental factors simultaneously, increasing the risk of certain malformations in men by as much as 50% [27]. We agree that UV EDCs can significantly influence male infertility. Our study primarily investigated sperm motility and semen parameters in relation to vitamin D levels. Further studies including environmental factors (such as UV exposure, drugs, or dietary habits), their impacts on sperm and semen parameters, and their association with vitamin D should be carried out.

## 6. Conclusions

We appreciate Dr. Abid et al.’s constructive feedback and the opportunity to discuss our study in more detail. The points raised are valid and provide avenues for future research. We remain confident in our finding that vitamin D levels are an important factor in male infertility and hope that our study serves as a foundation for further investigations in this area.
